# Antimonoid

**Published:** 1871-01

**Authors:** 


Antimonoid.—A welding power named antimonoid, has been in use for some time past in Germany, and found to be of great efficiency. The formula for its preparation has, until lately, been kept a secret; but we now learn that it consists of four parts of iron turnings, three parts of borax, two parts of borate of iron, and one of water.

A dentist in Philadelphia has traced out the career of 1,000 dentists, with this result: 163 died before they reached middle life, 643 attained fair success, 57 made fortunes, 27 died from intemperance and other vices, 96 failed entirely, and 3 committed suicide.

